# Biomarkers of Dietary Polyphenols in Cancer Studies: Current Evidence and Beyond

**DOI:** 10.1155/2015/732302

**Published:** 2015-06-09

**Authors:** Jincheng Wang, Lili Tang, Jia-Sheng Wang

**Affiliations:** Department of Environmental Health Science, College of Public Health, University of Georgia, Athens, GA 30602, USA

## Abstract

Polyphenols, commonly contained in fruits and vegetables, have long been associated with a protective role against multiple diseases and adverse health effects. Generally, *in vitro* and animal experiments have provided strong positive evidence, whereas evidence from *in vivo* and human epidemiological studies is not strong enough. Most epidemiological studies to date use food frequency questionnaire based dietary intake estimations, which inevitably incur imprecision. Biomarkers of polyphenol have the potential to complement and enhance current studies. This review performed a literature search of all epidemiological studies or controlled clinical/intervention trials which employed biomarkers of exposure for polyphenols to help assess their anticarcinogenic role, using studies on green tea polyphenols as a study model. Currently, studies on this topic are still limited; breast cancer and prostate cancer were the only widely studied cancer types. Isoflavone is the only widely studied polyphenol. In addition to associations between polyphenols and cancer risks, factors such as host genetic susceptibility, epigenetic modification, and gut microbiome patterns may also impact on the protective roles of polyphenols. More evidence should be collected by utilizing biomarkers of exposure for polyphenols in future epidemiological studies before a clear conclusion can be made.

## 1. Introduction

Extensive evidence has documented diets rich in fruits and vegetables protect human against multiple chronic diseases. Polyphenols, common constitutes in fruits and vegetables, are suggested to play a major protective role in disease prevention [1]. Evidences on beneficial effects of polyphenols are mainly derived from* in vitro* or animal experiments, as well as some human epidemiological studies [2]. Currently, epidemiological studies tend to accept a protective role of polyphenol containing foods on cardiovascular disease [3, 4]. Preventive effects of polyphenols on other chronic diseases, such as cancer, are generally less convincing [3, 5], with the exception of green tea polyphenols [6–8].

Part of the difficulties in assessing the beneficial effects of polyphenols in* in vivo* or epidemiological studies is related to poor understanding of their bioavailability and metabolism in human physiological system [5]. The bioavailability of typical polyphenols is rather low upon oral administration, due to their poor water solubility, rapid biotransformation, membrane permeability, and molecular size [9, 10]. In addition, most* in vitro* or animal studies examined aglycones of polyphenols [11]; however, most polyphenols exist as glycosides in natural food sources [3, 12], which are known to be less bioactive as compared to their aglycones [3, 11]. Moreover, although human gut microbiota is capable of transforming polyphenol glycosides into their aglycone, intestine barrier and first-pass metabolism will transform polyphenols into methoxylated, glucuronidated, sulfated, or other conjugated forms [4]. Therefore, aglycones of polyphenols rarely exist in systemic circulation [12].

Another difficulty in assessing the role of polyphenol in* in vivo* and epidemiological studies is the lack or inaccuracies in the measurement of bioavailable and effective polyphenols, which may lead to weaker or even spurious correlations between exposure and disease risks [5, 13]. In epidemiological studies, food frequency questionnaire (FFQ) based estimation of dietary intake of specific polyphenols is the most commonly used surrogate of polyphenol exposure. This method suffers from imprecision [14, 15]. Since the mid-1990s, the analytical methods for specific polyphenolic compounds in body fluids have become available [14]. Biomarkers as a result of these developments, such as measurement of urinary excretion or plasma level of polyphenols, could complement or even replace questionnaire based dietary assessment [2, 4–6].

There have been numerous reviews and meta-analysis on the chemopreventive effects of polyphenols on different cancer types [16–24]. All these reports concluded that existing evidence is insufficient to support a chemopreventive role of polyphenols. However, studies reviewed or summarized within abovementioned reports are mostly questionnaire based. The purpose of this review is to focus on epidemiological studies which employed biomarkers of polyphenol exposure, using green tea polyphenols as an example, and to examine whether using more accurate measurements of polyphenol biomarkers could reveal the beneficial role of polyphenol compounds as suggested in* in vitro* and animal studies. This review will start from a brief summary of bioavailability studies on common polyphenol compounds that have been suggested to have an anticarcinogenic role, followed by a detailed summary of epidemiological studies or clinical trials using polyphenol biomarkers and a summary of studies on green tea polyphenols as a study model.

## 2. Bioavailability of Common Polyphenols

A previous review [25] has comprehensively examined bioavailability and bioefficacy of polyphenols by summarizing 97 studies. Based on that report and new evidences thereafter, we briefly discuss the human dietary intake, the absorption, and pharmacokinetics of common polyphenols. The reason of summarizing bioavailability studies is that biomarkers of polyphenol exposure are most likely to be either the direct bioavailable forms of a specific polyphenol or their metabolites. Therefore, understanding the bioavailability of these compounds could help us assess the validity of biomarkers applied in existing studies and suggest potential biomarkers.

### 2.1. Anthocyanins

Anthocyanins are one of the most widespread polyphenols and exist in large amount in some diets [25]. An earlier estimate suggested that the daily intake of anthocyanin can be as high as 215 mg/day/person [26]. More recent reports have suggested a lower but still substantial dietary intake. In a study in Finland, FINDIET 2002 Survey, mean dietary intake of anthocyanins was estimated to be 47 mg/day/person [27]. Another report based on the SU.VI. MAX study in France estimated mean dietary intake of anthocyanins to be 57 mg/day/person [28]. An estimate from the European Prospective Investigation into Cancer and Nutrition (EPICN) Spanish subcohort reported a much lower intake of about 18.88 mg/day/person [29]. This estimate is in the same magnitude of another study conducted among US adults (12.5 mg/day/person) [30].

Previous evidence has shown that anthocyanins are absorbed as intact glycosides or as metabolized conjugates in stomach or intestine [31, 32]; no aglycones could be recovered either in plasma or in urine [33]. Animal and human studies further demonstrated that anthocyanins could be rapidly absorbed [33]; the plasma concentration reaches its peak concentration in 0.5–4 hours in both human and animal studies [31]. However, the bioavailability of anthocyanins is very low, with a majority of studies reporting the urinary recovery less than 1% [33, 34]. Some recent studies have argued that the bioavailability of anthocyanins may not be as low as it is used to be believed because a variety of metabolites of anthocyanins may be absorbed in gastrointestinal tract [31].

### 2.2. Flavonols/Quercetin

Flavonols are another group of widely distributed polyphenols. Quercetin is one of the most commonly dietary available flavonols, which can be found in onions, tea, and wine. The daily intake of quercetin, however, might be low because of relatively low dietary content [25]. Average dietary intake of quercetin in a Netherland study is 16 mg/day/person [55]. More recent reports from other European studies have shown daily intakes of flavonols to be 5.4 mg/day/person in Finland [27], 51 mg/day/person in France [28], and 18.7 mg/day/person in Spain [29]. A recent US study based on NHANES 1999–2002 estimated the average daily intake of flavonols to be 12.8 mg/day/person [56].

Although earlier researchers used to believe that quercetin cannot be absorbed by humans [25], Hollman et al. firstly showed that quercetin could indeed be absorbed in small intestine as mainly glucosides (52% of absorbed quercetin) and as quercetin aglycone (24%) or quercetin-3-rutinoside (17%) [55]. More recent studies [57–59] confirmed this finding, but current consensus is that quercetin aglycone cannot be absorbed into systemic circulation and it is quercetin glucuronides instead of glucosides that are present in plasma or urine after oral intake of quercetin. Graefe et al. found that different sugar moiety of quercetin glycosides influenced their bioavailability [58]. For example, glucosides of quercetin (the major form in onions) could be rapidly absorbed, and the plasma quercetin reached its peak concentration, 2.1–2.3 *μ*g/mL (about 100 mg equivalent quercetin intake), about 0.7 hours after oral administration. But rutin forms of quercetin (the major form in tea) were slowly absorbed, reaching its peak concentration, 0.3–0.6 *μ*g/mL (100 mg equivalent quercetin intake), about 4.3 to 7 hours after administration with a much lower relative peak concentration. Urinary recovery of quercetin is low: 48-hours after oral intake, about 6.4% of quercetin was recovered if taking glucosides, but less than 1% could be recovered if taking rutin form [58].

### 2.3. Flavanones

Flavanones represent a small group of polyphenols. They almost exclusively exist in citrus fruits and in some aromatic herbs such as mint [25]. Although dietary sources of flavanones are limited, their daily intakes can be high because citrus fruits are commonly consumed in different forms [60]. In a study in Finland, hesperetin, a major flavanone compound, was reported to be the most highly consumed flavonoid [61]. Other European studies have reported the daily intake of total flavanones (including eriodictyol, hesperetin, and naringenin) to be 27 mg/day/person in Finland [27], 50 mg/day/person in Spain [29], and 26 mg/day/person in France [28]. The study among US adults reported daily intake of 13.3 to 15.5 mg/day/person [56].

Among a few studies on the bioavailability of flavanones in human subjects, Manach et al. conducted a crossover study among five healthy volunteers who consumed two different doses of orange juice and reported that no free flavanone aglycone existed in plasma after each dosing [60]. Further analysis suggested that plasma flavanone is glucuronidated or sulfoglucuronidated [60]. The two compounds, hesperetin and naringenin, tested in this study were absorbed much slower and at much lower level as compared to other previously mentioned polyphenols. Plasma hesperetin reached its peak concentration, about 0.14 *μ*g/mL after 222 mg oral intake and 0.39 *μ*g/mL after 440 mg oral intake, about 5-6 hours after administration. Naringenin reached its peak concentration, about 0.054 *μ*g/mL after 96 mg intake and 0.016 *μ*g/mL after 48 intake, about 5.4 hours after administration [60]. In addition, about 4–6% of hesperetin intake and about 7% of naringenin intake could be recovered from urine, and not surprisingly both compounds were in glucuronidated or sulfoglucuronidated form [60].

### 2.4. Isoflavones

Isoflavones are only found in soybean-derived products [25]. The natural isoflavones, such as daidzein or genistein, are usually in glucose-conjugated form [62]. The intake of isoflavone also varies greatly by different countries. The recent report among US adults estimated the daily intake of isoflavones was the lowest among all flavonoids, only about 1.2 mg/day [56]. Similar results were obtained from a Finnish study (0.9 mg/day) [27] and nearly none in a Spanish study [29], whereas in Asia, a study in Japan [63] among 1232 healthy participants has reported that the median intake of daidzein was from 9.5 to 12.1 mg/day and the intake of genistein was from 14.9 to 19.6 mg/day, more than ten times as compared to western countries. The median intake of isoflavone in another study [64] among 60 healthy Chinese women was as high as 39.3 mg/day.

The bioavailability of isoflavones is well documented. An early study [65] recruited eight volunteers and measured the plasma isoflavones concentrations after two doses (high and low) of either isoflavone aglycone or glycosides. This study found that plasma isoflavones reached peak concentrations 2–4 hours after intake of aglycone, which was faster than correspondent glycosides (about 4–8 hours after intake); the peak concentrations for aglycones were also higher than glycosides. Based on these findings, it was concluded that isoflavone aglycones were more efficiently absorbed than glycosides. Another study [66] revealed similar pattern, although much slower: for genistein and daidzein glycosides (genistein and daidzein), the mean time of reaching peak plasma concentration was around 9 hours, whereas for genistein and daidzein aglycone, the mean time was around 5-6 hours. The peak concentrations measured in this later study for daidzein and genistein were 194 ng/mL and 341 ng/mL, whereas the peak concentrations for daidzein and genistein were 394 ng/mL and 341 ng/mL. These findings further suggested that the absorption of glycosides was higher as compared to their respective aglycones based on the area under pharmacokinetic curves [66]. The discrepancy between these two studies might be because the first study [65] did not measure isoflavone level between 6 and 24 hours; it was possible that the glycosides levels were still rising after 6 hours and therefore were not captured.

The longer time for isoflavone to reach a peak plasma concentration after intake of glycosidic form was attributed to the fact that glycosides cannot be directly absorbed in intestine in both abovementioned studies; they have to be firstly hydrolyzed [65, 66]. A later study confirmed that no glycosides could be absorbed by human volunteers and found that the circulating forms of isoflavone in plasma were mainly conjugated forms [12]. The urinary excretion of isoflavone is typically high. As shown in one study [67], 30% of daidzein and 9% of genistein could be recovered in urine after a single dose of each isoflavone among healthy premenopausal women.

### 2.5. Flavanols/Catechins

Catechins, one of a major group in flavanols or flavan-3-ols, are the principle components in tea, apple, chocolate, pear, grape, and red wine [25]. Since its discovery more than 5000 years ago, tea has grown to be the most widely consumed beverage in the world [13]. The habit of tea consumption varies by different geographic regions: black tea is principally consumed in Europe, North America, and North Africa while green tea is consumed throughout Asia. Oolong tea is particularly consumed in China and Southeastern Asia [68]. The three classes of tea were produced by different manufacturing processes: green tea without fermentation, oolong tea with partial fermentation, and black tea fully fermented. Fermentation converts tea polyphenols into theaflavins, thearubigins, theasinensins, and proanthocyanidin polymers that are abundant in black tea [69]. Green tea is most abundant for tea catechins [70] which include (−)-epicatechin (EC), (−)-epigallocatechin (EGC), (−)-epicatechin gallate (EG), and (−)-epigallocatechin gallate (EGCG) [13]. Due to the long history of tea drinking and its long recognized beneficial effects, catechins are probably the most extensively studied polyphenols.

The recent report in US [56] estimated that the daily consumption of flavan-3-ols was the highest among all flavonoids, as high as 156.5 mg/day among US adults. The European studies reported average flavan-3-ols intake to be 15.67–22.06 mg/day in Spain [29] and 99 mg/day in France [28]. Although intakes of flavanols by Asian populations are speculated to be very high, there is few quantitative data reporting the amount of their flavanols daily intakes available.

Multiple lines of evidence have documented bioavailability of tea catechins. A human study found that after intake of about 1.2 g decaffeinated green tea (DGT, containing 88 mg of EGCG, 82 mg of EGC, 33 mg of ECG, and 32 mg of EC), EGC, EGCG, and EC, but not ECG, were detected in volunteers plasma samples [71]. However, more recent studies did detect ECG after dosing either pure ECG or green tea to healthy volunteers [72, 73]. The discrepancy might be because of the different conditions of analytical methods to detect ECG [72]. Regarding the chemical speciation of catechins after intake of tea polyphenols, EGC was originally reported to present mostly in glucuronidated form (57–71%) and sulfate form (23–36%); only a small portion (3–13%) of free form was presented, whereas EC was exclusively in the conjugated form, with approximately 67% as glucuronidated form and 33% as sulfated form [71]. These results were confirmed by a more recent study [74]. For EGCG, a more recent study [74] found that EGCG were presented mostly in its free form.

Yang et al. [13] demonstrated that, after ingestion of DGT, plasma concentrations of EGCG, ECG, and EC reached their peak concentrations about 1.5–2.5 hours after intake [13]. The plasma concentrations for EGCG, ECG, and EC were found to be dose dependent at 1.4–5.5 hours after ingestion [13]. The peak concentrations of EGCG, EGC, and EC after intake of 3.0 g DGT were 0.326 *μ*g/mL, 0.508 *μ*g/mL, and 0.189 *μ*g/mL [13], suggesting that EGCG has a lower absorbability. These results were confirmed by later studies [72–74]. In addition, multiple evidence has showed that no EGCG could be detected in urine after oral intake of catechins [13, 71, 72, 74]. One possible explanation is that EGCG or its metabolites are mainly excreted in bile as found in rat [13].

## 3. Epidemiological Studies Employing Biomarkers of Polyphenol Exposure

For the search of epidemiological studies or controlled clinical trials which employed biomarkers of polyphenol exposure to assess their anticarcinogenic role, we included all available literature from articles of original research to meeting proceedings since 1995 because analytical techniques have been available since the mid-1990s [14]. We also performed a manual search using reference lists of original articles, meta-analysis, and relevant reviews. Out of 315 articles, 20 were deemed relevant to the scope of this review after carefully examining the titles and abstracts and therefore summarized. Information on the 20 articles is presented in Table 1.

Among the 20 articles, the vast majority of studies (16 out of 20) assessed the beneficial effects of isoflavone using either plasma isoflavone concentrations or urinary excretion as biomarkers of exposure (Table 1). This might partly be because plasma and urinary excretion of isoflavone have been recognized as valid biomarkers of exposure [44, 46, 75] while other polyphenols remain to be elicited. The other three studies assessed the effects of tea catechins. A recent biomarker validation study confirmed that blood ECG and EGCG could reflect the diet intake of green tea polyphenol and therefore suggested their use in population level studies [6].

Breast cancer (8 out of 20) and prostate cancer (6 out of 20) are the most frequently studied cancer types; two other studies examined colorectal cancer; three examined gastric cancer; one examined liver cancer; and one examined lung cancer. It was also interesting to see that the majority of the studies were focused on Asian population (10 out of 20) and European population (6 out of 20); only two studies were conducted in United States; another one was conducted in Jamaica and one in Australia.

### 3.1. Breast Cancer

All eight studies assessed the role of isoflavone on breast cancer risk. Urinary excretion rates of daidzein and its metabolite equol were used as biomarkers of isoflavone exposure to assess the risk of breast cancer among a group of Australian women [35]. Increased excretion of equol (indicating a higher bioavailable amount) was reported to be significantly associated with a reduced risk. Subjects among the highest quartile of equol excretion had a 73% lower risk of developing breast cancer as compared to reference level (lowest quartile) (odds ratio (OR_high  versus  ref._) = 0.27, 95% confidence interval (CI) was 0.10–0.69); the dose-response relationship was also significant for equol (*P*-trend = 0.009). However, no significant association with increased daidzein excretion (OR = 0.47, 95% CI = 0.17–1.33) was found [35]. Nevertheless, two European studies [36, 37] failed to find a significant association between isoflavone exposure and breast cancer risk, although the risk tended to be reduced. Another study [38] reported that higher plasma genistein level (but not daidzein) was associated with a significantly reduced risk (OR_high  versus  ref.  _ = 0.68, 95% CI 0.47–0.98). One possible reason for the null association suggested by these authors is that the intakes of isoflavone among European population were mainly from dietary additions and therefore relatively low [36, 38]. It is true that the urinary excretions from all these studies were below 1.0 nmol/mg creatinine for isoflavones and the median plasma levels were all below 10–15 nmol/L. Other possible reasons might be the biases employed due to the study design. For example, in one study [37], blood samples were taken after the cases were diagnosed; the median time between subject participation (when blood samples were taken) and diagnosis was 2 months. It was possible that patients might have changed their diet after diagnosis.

Moreover, a positive association was reported when examining EPICN-Norfolk (UK) subcohort using both plasma level and urinary excretion of isoflavones; exposure to all isoflavone was associated with increased breast cancer risk, significantly so for daidzein and equol [39]. In addition to the consistently low levels of isoflavone among study subjects as in other European countries, interpretation of results from this study should be cautious. A log_2_ OR was reported in this study [39], willing to present the relative risk associated with doubling of the exposure. However, their calculation might be problematic because log_2_ transformation should be made on exposure variable (plasma or urinary level of isoflavones), not the OR itself [76].

Three studies among Asian populations reported relatively consistent results [40–42]. In a study using Shanghai Breast Cancer Study cohort samples, a lower but insignificant risk between urinary excretion of daidzein and genistein and breast cancer risk was found [40]. A relative small sample size (60 cases and 60 controls) may contribute to the results. A later study from the same group [41], using more samples from 250 cases and 250 controls and a more sensitive technique (LC/MS instead of spot urine analysis), found significant lower risk from subjects with the highest tertile of urinary daidzein level (OR_high  versus  ref._ = 0.54, 95% CI = 0.34–0.85). A significant dose-response relationship was also found (*P*-trend < 0.01). Another study with Japan Public Health Center cohort samples reported that plasma genistein concentrations were significantly associated with breast cancer risk and OR_high  versus  ref._ = 0.34 (95% CI = 0.16–0.74) [42].

### 3.2. Prostate Cancer

Isoflavone levels were measured in five of the six studies assessing the prostate cancer risk with dietary intake of polyphenols. In a nested case-control study [43] with the previously mentioned Japan Public Health Center cohort samples, highest tertile for equol exposure was significantly associated with a decreased risk of total prostate cancer (OR_high  versus  ref._ = 0.60; 95% CI = 0.36–0.99; *P*-trend = 0.03); this association was even stronger in localized cases (OR_high  versus  ref._ = 0.43; 95% CI = 0.22–0.82; *P*-trend = 0.02). Plasma genistein level also tended to be inversely associated with total prostate cancer (OR_high  versus  ref._ = 0.54; 95% CI = 0.29–1.01; *P*-trend = 0.03). However, the association could not be seen in advanced cases.

Similar to breast cancer, the association between dietary isoflavone intake and prostate cancer risk was not obvious in several European studies. A study using EPICN-Norfolk subcohort samples with 193 cases and 828 controls did not find any association between the plasma or urinary biomarkers for isoflavone exposure and prostate cancer risk [44]. Another study using the whole EPICN cohort samples with 950 cases and 1042 controls also failed to find sufficient evidence, although plasma genistein levels tended to be associated with reduced risk (OR_high  versus  ref._ = 0.74; 95% CI = 0.54–1.00) [45]. However, another nested case-control study with samples from multiethnic population in Hawaii and California found that urinary excretion of daidzein was associated with reduced risk of prostate cancer (OR_high  versus  ref._ = 0.55; 95% CI = 0.31–0.98) [46]. The Jamaican study [47] analyzed spot urinary isoflavones and also found urinary equol level was significantly associated with reduced risk of total prostate cancer (OR_high  versus  ref._ = 0.48; 95% CI = 0.26–0.87) and high-grade disease (OR_high  versus  ref._ = 0.29; 95% CI = 0.13–0.60). No associations between urinary excretion of daidzein and genistein and the risk of prostate cancer were observed.

The only study that assessed the beneficial effects of polyphenols other than isoflavone is a clinical trial conducted in US [48]. This randomized, double blinded, and placebo controlled trial studied polyphenol E (a drug that contains EGCG) in men with prostate cancer scheduled to conduct radio prostatectomy. Volunteers were dosed with polyphenol E that contains about 800 mg EGCG, and no significant reduction of prostate cancer tissue biomarker was found and EGCG levels in prostate tissue were rather low.

### 3.3. Colorectal Cancer

Only two studies assessing colorectal cancer risk adopted biomarkers of polyphenol exposure. In a nested case-control study with Shanghai cohort samples [49], the effects of tea catechins were assessed, and individuals with high prediagnostic urinary EGC levels and 4′-MeEGC were associated with reduced risk of colon cancer. OR_high  versus  ref._ = 0.40 (95% CI, 0.19–0.83) for EGC; a dose-response trend was also presented (*P*-trend = 0.02); OR_high  versus  ref._ = 0.41 (95% CI, 0.20–0.84; *P*-trend = 0.006) for 4′-MeEGC. No association between tea polyphenols and rectal cancer risk was observed.

In the previously mentioned EPICN-Norfolk subcohort, no associations were observed between plasma or urinary levels of daidzein, genistein, and equol and colorectal cancer risk [44].

### 3.4. Gastric Cancer

Studies on beneficial effects of polyphenol on gastric cancer risk were mainly conducted in Asian countries, probably because Eastern Asia region has the highest gastric cancer incidence worldwide [77]. A study using the previous mentioned Shanghai cohort samples found that urinary tea catechin (EGC) positivity was associated with reduced risk of gastric cancer (OR = 0.52; 95% CI = 0.28–0.97) [50]. Another nested case-control study in South Korea [51] also found significant reductions in gastric cancer risk: OR_high  versus  ref._ = 0.21 (95% CI = 0.08–0.58) for daidzein, OR_high  versus  ref._ = 0.54 (95% CI = 0.31–0.93) for genistein, and OR_high  versus  ref._ = 0.50 (95% CI = 0.27–0.90) for equol, respectively [50]. Another Japanese study [52], however, failed to find such a significant relationship.

### 3.5. Other Cancers

In a study of the association between tea catechins and esophageal cancers conducted in China, no significant association was found [50]. A Japanese nested case-control study [53] assessed the relationship between isoflavone and lung cancer and found that plasma genistein concentration was significantly associated with reduced risk of lung cancer (OR_high  versus  ref._ = 0.31; 95% CI = 0.12–0.86). Daidzein and equol, however, did not present similar associations. The association between dietary intake of tea polyphenols and liver cancer risks will be summarized in Section 4 as part of illustration of green tea polyphenol study model.

### 3.6. Summary

Currently, studies employing measurements of body fluid polyphenol concentration as biomarkers of exposure to assess the protective roles against cancer risk are still very limited. Breast cancer and prostate cancer are the most commonly studied cancer types. It is interesting to find that most studies among Asian populations presented a reduced risk, whereas studies in other parts of the world are still inconsistent. One probable reason for this striking contrast might be because Asian populations ingest a much higher amount of isoflavones as compared to other regions, which was evidenced in the studies summarized and data from Table 1.

Most of the studies summarized here adopted case-control design, which usually employs a single measurement for evaluation of efficacy, which may not accurately reflect the actual continuous exposure. The analytical methods and sampling procedures employed in this type of studies may also have some limitations; for example, in all isoflavones related studies, samples were analyzed with pretreatment with *β*-glucuronidase and sulfatase which transformed nearly all conjugated forms into their respective aglycones. However, as previously mentioned, conjugated forms of polyphenol do not have the same effectivity as their aglycones; in fact, in most cases conjugated forms are less bioactive. In addition, some experimental conditions may have significant impacts on the study results. For example, time of sample collection is crucial; if samples are collected after the meal, the concentrations are usually higher than those collected in the early morning [49].

## 4. Green Tea Polyphenol as a Study Model

With strong evidence from* in vitro* and animal studies as well as historical perspective, but relatively less convincing evidence from previous epidemiological studies, green tea polyphenol (GTP, mainly catechins) attracts much attention. To bridge discrepancy between animal data and human studies, simultaneously accounting for the limitations in epidemiological studies and variations in individual response, a series of human perspective studies incorporating biomarkers as surrogate endpoints have been conducted to elucidate GTP's role in human health with better accuracy and precision and therefore are summarized here as a study model.

### 4.1. Phase II Clinical Trial with Green Tea Polyphenols in Southern Guangxi, China

A randomized, double blinded, and placebo controlled, phase IIa chemoprevention trial was conducted in Guangxi, China, to validate GTP biomarkers in human body fluids, study the modulative effects of GTPs on carcinogen biomarkers, and directly examine the possible adverse effect of GTPs in human subjects [78, 79]. The study screened 1,200 blood samples for hepatitis B virus (HBV) and aflatoxin (AF) biomarkers and recruited 124 residents who were both HBsAg and AF-albumin adducts positive, aged 20–55 with normal liver function test, serum alpha-fetoprotein negative, no personal history of cancer, and no use of prescribed medications. These subjects were regarded as having higher risk of liver cancer. Two doses of GTP (500 mg and 1000 mg, equivalent to two and four cups of tea drink, resp.) and placebo were administrated to three groups. Initial studies did not find significant differences on adverse effects and parameters representing liver and kidney function among three groups, indicating the relative safety of GTP in human subjects [78, 79].

### 4.2. Validation of Green Tea Polyphenols Biomarkers in Human Population Levels

Several follow-up studies [6, 80, 81] were conducted to validate the use of GTP biomarkers in human studies based on previously mentioned phase II clinical trial. A total of 340 urine samples collected at day 0 (baseline) and 1 and 3 months of the clinical trial were analyzed. Urinary excretion of GTPs was adjusted by creatinine levels. Trace amounts of GTP components were detectable for all 3 groups at baseline with no statistical significance (*P* = 0.92). Analysis of urine samples collected at 1 and 3 months revealed that levels of urinary EGC and EC were dose-dependently elevated in GTP-treated groups. Significant differences between times and groups of treatment (*P* < 0.05) were also found. A total of 343 plasma samples collected at the same time points were analyzed. Trace amounts of GTP components were detectable for all 3 groups at baseline with no statistical significance (*P* > 0.33). Analysis of samples collected at 1 and 3 months revealed a similar pattern as in urine sample: plasma levels of EGCG and ECG were dose-dependently elevated in GTP-treated groups. Significant differences between times and groups of treatment (*P* < 0.05) were also found. These results demonstrated that urinary excretion of EGC and EC and plasma levels of EGCG and ECG can serve as valid biomarkers for green tea consumption and may be applicable in future intervention and epidemiologic studies [81]. Luo et al. further confirmed previous results by a metabolomics study in which 106 metabolites of GTP were identified, and 56 of them were chosen to construct discriminant functions (DFs) based on the data at 1 and 3 months. Overall this study found metabolic profiles effective in discriminating different GTP dosages [80].

### 4.3. Modulation of Carcinogen Biomarkers by Green Tea Polyphenols

Reactive oxygen species (ROS), produced in the process of carcinogen metabolism, inflammation, and aerobic respiration, can attack all macromolecules including lipids, proteins, and DNA. Urinary 8-hydroxydeoxyguanosine (8-OHdG) generally reflects the whole body's oxidative DNA damage and repair and becomes a putative biomarker for oxidative stress [82]. Detection of urinary 8-OHdG provides a sensitive and noninvasive means to evaluate the efficacy of chemoprevention. Urinary excretion of GTPs and 8-OHdG was therefore analyzed in samples collected from the phase II clinical trial [54]. At the end of 3-month intervention, 8-OHdG levels decreased significantly in both GTP-treated groups, suggesting that clinical intervention with GTPs was effective in diminishing oxidative DNA damage [54].

The mediation effects of GTP among subjects with positive HBsAg and AF-albumin adducts (high liver cancer risk subjects) were also assessed in above phase II clinical trial [83]. The AF-albumin adduct levels in serum samples collected at 1 month of the intervention were significantly decreased in the high-dose group (*P* < 0.05) as compared with level in the placebo group. The levels of AF-albumin adducts in serum samples collected at 3 months of the intervention showed significant decrease in the low- and the high-dose groups as compared with the levels of the baseline values (*P* < 0.05). Levels of AFM_1_ and AFB_1_-mercapturic acid (AFB_1_-NAC) in urine samples collected at 3 months of the intervention showed that treatment of GTP for up to three months significantly decreased levels of AFM_1_ in both low-dose and high-dose intervention groups. Treatment of GTP elevated levels of AFB_1_-NAC in GTP-treated groups [83]. These results demonstrate that treatment with GTP effectively inhibits phase I metabolic enzyme activities and induces phase II metabolic enzyme activities.

### 4.4. Long-Term Clinical Trial with Green Tea Polyphenols in Southern Guangxi, China

Current short-term evidence showed that taking GTPs does not have obvious negative effects, elevates plasma and urinary GTP components, and mediates oxidative stress and biomarkers of high liver cancer risk. Long-term clinical trial was therefore warranted. Yu et al. [84] initiated a 3-year randomized, double blinded, and placebo control study in 2004. This study screened 10,000 adult people and recruited 1,826 HBsAg positive adults with normal liver function test, negative serum alpha-fetoprotein, no personal history of cancer, and no use of prescribed medications, in Guangxi, China. Two capsules (500 mg GTP or placebo) were instructed to be taken twice daily after meal. The efficacy of the trial is measured as the ability of GTPs to decrease the levels of serum and urinary AF biomarkers and urinary 8-OHdG within the first year. The efficacy of GTPs will be eventually measured as the ability to reduce actual incidence of hepatocellular carcinoma (HCC) in the studied population at the end of three years of intervention. The HCC incidence rate for years 2 and 3 was significantly reduced in GTPs-treated group (443.46/100,000 person year) as compared to that of placebo group (1092.39/100,000 person year) (*P*_one-sided_ = 0.039) [84].

## 5. Prospective

### 5.1. Interplay between Dietary Intakes, Genetic Factors, Epigenetic Mechanisms, and Postgenomic Considerations

Adopting biomarker-based methods to improve the accuracy could complement and enhance studies on the beneficial role of dietary polyphenol on human health. This method allows researcher to gain more insights on specific polyphenol compounds on certain diseases. For example, equol, the metabolite for daidzein, was considered the most bioactive isoflavone but could not be accurately measured using traditional methods [85]. Although more knowledge has been obtained since involving biomarkers of exposure for polyphenol intake, there are still many questions regarding the relationship between polyphenols and cancer risks waiting to be answered, from the perspectives of large interpersonal and interethnic variations in absorption, metabolism, and excretion of polyphenols [85]. For example, genetic variation on metabolizing enzymes may play important roles in determining the reduced risks of polyphenols, as evidenced by several studies [86–91]. Besides, epigenetic regulation in physiological environment has been widely considered to play an important role in biological functions. Their potential role in the relationship between polyphenol and cancer risk has also been suggested [92]. For example, Fang et al. suggested dietary polyphenols may play a role in DNA methylation by inhibiting DNA methyltransferases [93].

From dietary intake, to biomarkers, to genetic and epigenetic factors, and even to metabolomics [94], all focused on the subject itself when assessing the role of polyphenol in disease prevention. This scheme has been challenged in recent years since people have gained more understanding of the environments surrounding and inside us. Gut microflora has long been suggested to play an important role in human metabolism of polyphenols; however their role on disease prevention is only recently suggested [94]. With the rapid development of high throughput second-generation or even third-generation sequencing techniques, researchers can now directly measure the interplay between dietary nutrients including polyphenols intakes and gut microflora changes: how its community structure changes (microbiome study) and how its function changes (bacteria metabolomics), which may lead to another round of scheme shift.

### 5.2. Future Directions

Given current evidences and potential opportunities, research works in following aspects are suggested to improve in the future.

- Epidemiological evidence on beneficial role of specific polyphenols on specific cancer risk should continuously be collected, especially on polyphenols such as tea polyphenols, in multiple cancer types with different stage of carcinogenesis.
- When measuring a specific biomarker, more attention should be paid to the chemical speciation of that biomarker.
- The metabolomics study should be conducted to screen more sensitive and useful biomarkers and generate hypotheses driven measurement. With these hypotheses, the interplays between genetic and epigenetic factors and polyphenols on cancer risk should be further examined.
- Microbiome changes in response to polyphenol exposure and their interplay with other factors should be elicited.

## 6. Conclusions

This review performed a literature search of all epidemiological studies or controlled clinical trials which employed biomarkers of exposure for polyphenols to help assess their anticarcinogenic role, using studies on green tea polyphenols as a study model. Currently, studies on this topic are still very limited. Breast cancer and prostate cancer were the only widely studied cancer types. Isoflavone is the only widely studied polyphenol. Protective roles of isoflavones against breast cancer and prostate cancer were consistently reported among Asian populations. Evidences on other populations and cancer sites are still insufficient. Prospective studies on green tea polyphenols have suggested a protective role against carcinogen biomarkers of liver cancer risks. In addition to associations between polyphenols and cancer risks, factors such as host genetic susceptibility, epigenetic modification, and gut microbiome patterns may also impact on the protective roles of polyphenols. More evidences should be collected by utilizing biomarkers of exposure for polyphenols in epidemiological studies and extended to molecular level before a conclusion can be made towards their protective roles.

## Figures and Tables

**Table 1:** Epidemiological studies/clinical trials employed biomarkers of polyphenol exposure.

Reference	Location	Types of study	Cancer type	Number of subjects	Polyphenol	Plasma level^1^ nmol/L	Urinary excretion^1^ nmol/mg creatinine^2^
[35]	Australia	Case-control	Breast cancer	Cases: 144 Controls: 144	Equol		nmol/24 h Cases: 97 Controls: 108.8
					Daidzein		nmol/24 h Cases: 782.9 Controls: 913.4

[36]	Netherland	Nested case-control	Breast cancer	Cases: 88 Controls: 268	Genistein		Cases: 0.70 Controls: 0.81

[37]	Germany	Case-control	Breast cancer	Cases: 220 Controls: 237	Genistein	Cases: 4.5 Controls: 3.7	

[38]	Netherland	Nested case-control	Breast cancer	Cases: 383 Controls: 383	Daidzein	Cases: 9.20–11.25 Controls: 10.31–12.86	

[39]	UK	Nested case-control	Breast cancer	Plasma Cases: 97 Controls: 187	Daidzein Genistein	All: 8.7 All: 12.9	All: 0.60 All: 0.23
				Urinary Cases: 114 Controls: 219	Equol	All: 0.4	All: 0.0036

[40]	China	Nested case-control	Breast cancer	Cases: 60 Controls: 60	Daidzein		Cases: 2.15 Controls: 108.8
					Genistein		Cases: 0.76 Controls: 2.17

[41]	China	Nested case-control	Breast cancer	Cases: 250 Controls: 250	Daidzein		Mean Cases: 4.95 Controls: 9.90
					Genistein		Mean Cases: 2.19 Controls: 4.37

[42]	Japan	Nested case-control	Breast cancer	Cases: 144 Controls: 288	Daidzein	Cases: 65.7 Controls: 70.4	
					Genistein	Cases: 487.7 Controls: 534.7	

[43]	Japan	Nested case-control	Prostate cancer	Cases: 201 Controls: 402	Daidzein	Cases: 145.5 Controls: 139.6	
					Genistein	Cases: 330.4 Controls: 319.0	
					Equol	Cases: 65.7 Controls: 70.4	

[44]	UK	Nested case-control	Prostate cancer	Cases: 193 Controls: 828	Daidzein Genistein	All: 9.8 All: 25.5	All: 0.51 All: 0.25
					Equol	All: 0.04	All: 0.009

[45]	EU	Nested case-control	Prostate cancer	Cases: 950 Controls: 1042	Daidzein Genistein	All: 4.3 All: 6.7	
					Equol	All: 0.8	

[46]	US	Nested case-control	Prostate cancer	Cases: 249 Controls: 404	Daidzein		Cases: 0.173 Controls: 0.291
					Genistein		Cases: 0.048 Controls: 0.078
					Equol		Cases: 0.005 Controls: 0.004

[47]	Jamaica	Case-control	Prostate cancer	Cases: 175 Controls: 194	Daidzein		Cases: 0.199 Controls: 0.221
					Genistein		Cases: 0.280 Controls: 0.273
					Equol		Cases: 0.072 Controls: 0.067

[48]	US	Clinical trial	Prostate cancer		Catechin		

[49]	China	Nested case-control	Colorectal cancer	Cases: 162 Controls: 806	EGC		Geometric mean Nondrinker: 1.11 Tea drinkers: −4.47
					4′-MeEGC		Geometric mean Nondrinker: 4.30 Tea drinkers: −17.40

[44]	UK	Nested case-control	Colorectal cancer	Cases: 221 Controls: 889	Daidzein Genistein	All: 8.7 All: 23.1	All: 0.53 All: 0.20
					Equol	All: 0.04	All: 0.002

[50]	China	Nested case-control	Gastric cancer	Cases: 190 Controls: 772	Catechins		Unclear

[51]	Korea	Nested case-control	Gastric cancer	Cases: 131 Controls: 393	Daidzein	Cases: 80.5 Controls: 131.2	

[52]	Japan	Nested case-control	Gastric cancer	Cases: 483 Controls: 483	Daidzein	Cases: ~161 Controls: ~155	
					Genistein	Cases: 17 Controls: 18	

[50]	China	Nested case-control	Esophageal cancer	Cases: 42 Controls: 772	Catechins		Unclear

[53]	Japan	Nested case-control	Lung cancer	Cases: 126 Controls: 252	Daidzein	Cases: 115.2 Controls: 125.1	
					Genistein	Cases: 266.4 Controls: 267.9	
					Equol	Cases: 11.6 Controls: 14.4	

[54]	China	Clinical trial	Liver cancer		Catechin		

^1^All values are median, unless otherwise noted.

^2^All units are nmol/mg creatinine, unless otherwise noted.
